# Laparoscopic versus open appendicectomy performed by adult general surgeons in pre-teenage years children: a single-centre experience

**DOI:** 10.1308/rcsann.2023.0044

**Published:** 2024-02-16

**Authors:** E Hannan, EMY Lim, G Feeney, L O’Brien, JC Coffey, C Peirce

**Affiliations:** ^1^ University Hospital Limerick, Dooradoyle, Ireland; ^2^ Children’s Health Ireland at Crumlin, Dublin, Ireland; ^3^ University of Limerick, Dooradoyle, Ireland

**Keywords:** Acute appendicitis, Laparoscopic appendicectomy, Open appendicectomy, Paediatric appendicectomy

## Abstract

**Introduction:**

The utilisation of laparoscopic appendicectomy (LA) in children remains contentious despite the well-recognised advantages of laparoscopic surgery. The purpose of this study was to compare intraoperative and postoperative outcomes in LA and open appendicectomy (OA) when performed by adult general surgeons outside specialist paediatric practice in younger children.

**Methods:**

A retrospective review of all patients under the age of 13 who underwent LA for suspected appendicitis over a two-year period was conducted. These were case-matched with an equivalent number of patients who underwent OA during the same period. Intraoperative and postoperative outcomes were compared.

**Results:**

Fifty-one patients underwent LA during the study period. Patient demographics were statistically equivalent with the OA cohort. A statistically significant longer median operating time (58 vs 49min) was noted in the LA group, but intraoperative outcomes were otherwise comparable. LA, when compared with OA, was associated with a significant improvement in postoperative length of stay (2 vs 3 days, *p *< 0.001), postoperative complication rate (0% vs 6%, *p *= 0.01), negative appendicectomy rate (3.9% vs 17.6%, *p *< 0.001) and 30-day readmission rate (0% vs 5.9%, *p *= 0.03). No patients in the LA group required conversion to open surgery.

**Conclusion:**

LA can be safely delivered by adult general surgeons to younger paediatric populations outside the setting of paediatric specialist practice, with statistically significant improvements in postoperative outcomes noted when compared with OA. These findings are of importance in the current healthcare context where adult general surgeons continue to perform the majority of paediatric appendicectomies.

## Introduction

Acute appendicitis (AA) is the most common paediatric surgical emergency.^1^ Traditionally, appendicectomy has been performed by open surgery until the advent of laparoscopic approaches in the 1980s.^1,2^ Laparoscopy is associated with a shorter length of stay (LOS), decreased analgesic requirements and reduced wound complications.^3–5^ However, the benefit of laparoscopic appendicectomy (LA) in the paediatric population compared with open appendicectomy (OA) remains controversial.^6,7^ A recent meta-analysis revealed no significant difference in postoperative complications between approaches in children, and criticisms of LA include an increased operative cost, longer operating time and increased rate of intra-abdominal abscesses (IAA).^5–7^ It has also been suggested that the small body size of young children makes laparoscopic surgery challenging and the proposed benefit of laparoscopic incisions over a small Lanz incision questionable.^8^ Thus, the uptake of LA has been slower for children compared with adult populations.^1–8^

It remains common in many healthcare services that AA in children is managed by adult general surgeons because of the lack of availability of paediatric surgeons.^8,9^ Although there has been a demonstrable increase in the utilisation of LA for children in the past decade, this has been largely observed in specialist paediatric surgery practice.^8,9^ Recent multicentre studies show that most paediatric appendicectomies performed by adult general surgeons are by open surgery.^9,10^ This contrasts with the otherwise widespread utilisation of laparoscopy for index operations in adults.^4,5,9^

Although many studies have compared LA and OA in children, few have examined outcomes in those performed by adult general surgeons outside paediatric specialist practice.^6–9^ Multiple studies have also examined children defined as patients either under the age of 16 or 18.^6–10^ In such groups, it is possible that many children in their mid- to late-teens may be close to the size of an adult, and the specific difficulties posed by LA in a smaller abdomen are not encountered.^6–9^ The purpose of this study was to compare intraoperative and postoperative outcomes in LA and OA when performed by adult general surgeons in children under the age of 13, thus largely focusing on children prior to or early in puberty. This information could be used to inform safe practice in healthcare services where paediatric emergency surgery is frequently delivered by adult general surgeons.

## Methods

### Study design and data collection

A retrospective review of patients under the age of 13 years who underwent appendicectomy for suspected appendicitis over a two-year period (July 2019–July 2021) was conducted in a tertiary referral university teaching hospital where adult general surgeons provide emergency surgical care to paediatric patients. Currently, it is more common in our institution for patients with AA in this age demographic to be managed by OA. All patients in this age group who underwent LA were identified by reviewing the operating theatre logbooks. These patients were matched with an equivalent number who underwent OA during the same period. Patients who underwent OA were selected by a simple randomisation process that blinded investigators to selection to avoid bias. Following this, an in-depth review of medical records, operation notes, discharge summaries, radiology reports and histopathology reports was performed for those included in the study. Data collected included baseline demographics, operating time, estimated blood loss, intraoperative findings, the presence of complicated appendicitis (defined as an intraoperative finding of perforated appendicitis), postoperative LOS, opioid use, postoperative complications and histological confirmation of appendicitis. Patients admitted for an elective or interval appendicectomy were excluded from the study.

### Surgical technique

All operations were performed either by a consultant general surgeon or a general surgical registrar. The choice of surgical approach was based on individual surgeon preference. LA was performed using a 3-trocar technique with standard adult laparoscopic instrumentation. A 12mm balloon port was placed subumbilical by Hasson technique and two 5mm ports were inserted under laparoscopic vision in the left iliac fossa and suprapubic region. A diagnostic laparoscopy was routinely performed to identify the cause for the patient’s symptoms, which included a small bowel walk and inspecting the ovaries in female patients. Following division of the mesoappendix, the appendix was divided at the base after placement of three Endoloops® (two proximal, one distal) and extracted via the subumbilical incision via specimen retrieval bag. OA was performed using either a Lanz or Gridiron muscle-splitting incision with the appendix ligated at the base using a non-absorbable suture, following which the appendix stump was buried using a purse-string suture.

### Ethics

All patient data were anonymised for the purpose of this study. No identifying information was retained by the authors or included in the article. Because this was a retrospective service evaluation involving anonymised data, ethics committee approval was not required.

### Statistical analysis

Statistical analysis was performed using IBM SPSS version 24 (SPSS Inc, Chicago, IL, USA). Non-parametric data were expressed as median with interquartile range and parametric data as a mean with standard deviation. Univariate analysis was performed using Student’s *t*-test or Mann–Whitney *U* test for continuous variables, and Fischer’s exact test for categorical variables. A *p*-value of <0.05 was considered statistically significant.

## Results

### Patient demographics

During the two-year study period, 51 patients under the age of 13 years underwent LA for AA. These were compared with 51 patients in the same age range who underwent OA for AA during the same period and who were randomly selected from the operating theatre logbooks to avoid selection bias. In the LA cohort, the majority of patients were male (59%, *n *= 30) with a median age of 10 years (range 6–12) and a median weight of 35.2kg (range 23.3–76.6); most had an American Society of Anaesthesiologists (ASA) grade of I (76.5%, *n *= 39). In the OA cohort, the majority of patients were also male (61%, *n *= 31) with a median age of 9 years (range 4–12) and a median weight of 28.7kg (19–42.1); again most had an ASA grade of I (88.2%, *n *= 45). No statistical significance was observed in baseline characteristics between the two cohorts (Table 1).

**Table 1:** Patient demographics

	LA group (*n *= 51)	OA group (*n *= 51)	*p*-value
Median age (years)	10 (range 6–12)	9 (range 4–11)	0.25
Male (%)	59 (*n *= 30)	61 (*n *= 31)	0.42
Female (%)	41 (*n *= 21)	39 (*n *= 19)	0.34
ASA I (%)	76.5 (*n *= 39)	88.2 (*n *= 45)	0.06
ASA II (%)	21.6 (*n *= 11)	11.8 (*n *= 6)	0.09
ASA III (%)	2.0 (*n *= 1)	0 (*n *= 0)	0.16
Median weight (kg)	35.2 (range 23.3–76.6)	28.7 (range 9–42.1)	0.18

ASA = American Society of Anesthesiologists; LA = laparoscopic appendicectomy; OA = open appendicectomy

### Preoperative imaging

In the OA group, 43.1% (*n *= 22) underwent a preoperative ultrasound scan, whereas 37.3% (*n *= 19) underwent an ultrasound scan in the LA group (*p *= 0.27). Ultrasound scanning was diagnostic of appendicitis in 31.8% (*n *= 7) of open cases who underwent preoperative imaging, compared with 5.2% (*n *= 1) in those who underwent LA (*p *= 0.049).

### Intraoperative outcomes

In the LA group, the median operating time was 58min (range 32–107) compared with 49min (range 20–98) in the OA group (*p *= 0.01). Blood loss was minimal overall, with a median of 10ml recorded in both cohorts. No laparoscopic cases required conversion to open surgery. The majority (96%, *n *= 49) of open procedures were performed via a Lanz incision, with the remainder performed via a Gridiron incision. A diagnostic laparoscopy was documented in all laparoscopic procedures. Nine patients (17.6%) had complicated appendicitis in the LA group, compared with eight in the OA group (15.7%). Wound closure was performed by absorbable sutures in all laparoscopic cases, whereas 15.7% (*n *= 8) of open cases were closed by nonabsorbable sutures or skin clips. The majority of open cases were performed by consultants (60.8%, *n *= 31), whereas most laparoscopic cases were performed by registrars (56.9%, *n *= 29). A drain was placed in four open cases (7.8%) and three laparoscopic cases (5.9%; *p *= 0.69). No adverse events related to laparoscopic port placement, such as bladder injury, were recorded. During the study period, two patients underwent a diagnostic laparoscopy for suspected AA in which the decision was made not to remove the appendix based on intraoperative findings. In both cases the operating surgeon decided that the appendix was not acutely inflamed and that there was alternate pathology present to explain the patient’s symptoms. In one case, the patient was found to have a terminal ileal congenital band adhesion that appeared to be causing subacute small bowel obstruction and was thus divided. In the other case, terminal ileitis was encountered. There were no open cases for suspected AA in which the surgeon decided to leave the appendix in situ. No alternate diagnoses for the patient’s symptoms were made in any open cases, and there were no cases during this time in which a paediatric patient underwent an open exploration where an intraoperative decision to not remove the appendix was made (Table 2).

**Table 2:** Intraoperative outcomes

	LA group (*n *= 51)	OA group (*n *= 51)	*p*-value
Median operating time (min)	58 (32–107)	49 (20–98)	**0.01**
Median blood loss (ml)	10	10	1
Complicated appendicitis (%)	17.6 (*n *= 9)	15.7 (*n *= 8)	0.39
Drain placement (%)	5.9 (*n *= 3)	7.8 (*n *= 4)	0.34
Conversion to open surgery (%)	0 (*n *= 0)	N/A	N/A
Nonabsorbable wound closure (%)	0 (*n *= 0)	15.7 (*n *= 8)	**0.01**

LA = laparoscopic appendicectomy; N/A = not applicable; OA = open appendicectomy

### Postoperative outcomes

The median postoperative LOS was 2 days (range 1–6) in the LA cohort and 3 days (range 1–7) in the OA cohort (*p *< 0.001). Pain scores on the first postoperative day, recorded using a 10-point visual analogue score, were higher in OA (median 6/10) compared with LA (median 5/10; *p *= 0.16). Postoperative opioid use was also higher in patients who underwent open surgery, with 72.5% (*n *= 37) of the OA cohort requiring opioids on the first postoperative day compared with 52.9% (*n *= 27) in the LA cohort (*p *= 0.04). No postoperative complications were recorded in the laparoscopic group, whereas six (11.8%) were recorded in those who underwent open surgery (*p *= 0.01). Of these complications, two were postoperative IAAs managed by intravenous antibiotics, two were postoperative ileus and two were superficial surgical site infections that were managed with oral antibiotics. The 30-day readmission rate was 5.9% (*n *= 3) in the OA group, whereas no patients in the LA group were readmitted (*p *= 0.03) (Table 3).

**Table 3:** Postoperative outcomes

	LA group (*n *= 51)	OA group (*n *= 51)	*p*-value
Median postoperative LOS (days)	2 (range 1–6)	3 (range 1–7)	**<0.001**
Median day one pain scores (scale 1–10)	5	6	0.16
Day one opioid use (%)	52.9 (*n *= 27)	72.5 (*n *= 37)	**0.04**
Overall complication rate (%)	0 (*n* = 0)	11.8 (*n *= 6)	**0.01**
IAA (%)	0 (*n *= 0)	3.9 (*n *= 2)	0.07
Ileus (%)	0 (*n *= 0)	3.9 (*n *= 2)	0.07
SSI (%)	0 (*n *= 0)	3.9 (*n *= 2)	0.07
30-day readmission (%)	0 (*n *= 0)	5.9 (*n *= 3)	**0.03**
Negative appendicectomy (%)	3.9 (*n *= 2)	17.6 (*n *= 9)	**<0.001**

IAA = intra-abdominal abscess; LA = laparoscopic appendicectomy; LOS = length of stay; OA = open appendicectomy; SSI = surgical site infection

### Histopathological findings

The histopathological negative appendicectomy rate was higher in open surgery (*n *= 9, 17.6%) compared with laparoscopic surgery (*n *= 2, 3.9%; *p *< 0.001). In the open cohort, histopathological diagnoses included AA (51%, *n *= 26), acute suppurative appendicitis (9.8%, *n *= 5), acute gangrenous appendicitis (5.9%, *n *= 3) and perforated appendicitis (15.7%, *n *= 8). In the laparoscopic cohort, patients were diagnosed histopathologically with AA (52.9%, *n *= 27), acute suppurative appendicitis (13.7%, *n *= 7), acute gangrenous appendicitis (11.8%, *n *= 6) and perforated appendicitis (17.6%, *n *= 9). No patients were diagnosed with an appendiceal neoplasm or malignancy.

## Discussion

In this retrospective comparative analysis, we observed that LA performed by adult general surgeons in younger paediatric patients is a safe and feasible means of managing AA that compares favourably with OA, with a statistically significant reduction in the overall complication rate, postoperative LOS, opioid use, 30-day readmission rate and negative appendicectomy rate. Lower postoperative pain scores were also observed in the LA cohort, probably as a result of the reduced incision burden allowing for a quicker recovery and earlier discharge. Fewer wound-related complications were observed in the LA cohort, whereas surgeons had a greater tendency to favour nonabsorbable wound closure for open cases, thus subjecting children to a further medical appointment to allow for the removal of sutures or clips. Despite an equivalent rate of perforation between the two cohorts, the incidence of postoperative IAA and ileus was lower in patients managed by LA and no patients required conversion to open surgery. Although a statistically significant difference in median operating time favouring OA was noted, it is difficult to argue that a reduction of 9min is truly clinically significant, particularly in the context of a slower recovery and higher complication rate.

It is interesting to note that the negative appendicectomy rate was significantly lower in patients who underwent LA compared with OA. This is despite more patients in the open cohort undergoing preoperative ultrasound scanning, of which a greater proportion were diagnostic of AA than in the laparoscopic cohort. This is perhaps unsurprising, as performing a diagnostic laparoscopy is recognised as a standard step in LA, especially when the diagnosis is made on history and clinical examination alone without the support of definitive radiology.^10^ In all laparoscopic cases, a diagnostic laparoscopy was routinely performed, which allowed for the potential identification of an alternate diagnosis that may explain the patient’s symptoms, such as ovarian or small bowel pathology. This allowed for two patients during the study period to both avoid a negative appendicectomy and to have the cause of their symptoms accurately diagnosed. This ability to thoroughly evaluate the intraperitoneal cavity is a distinct advantage of LA compared with access via an incision in the right iliac fossa, during which the patient may have an alternate diagnosis missed and may also be exposed to the risk of an unnecessary appendicectomy.^3^

The results suggest that the management of paediatric AA by the adult general surgeon outside paediatric specialist practice is best delivered by a laparoscopic approach, with less postoperative morbidity compared with OA. This is important information because the majority of children undergoing appendicectomy in the UK and Ireland do so in non-specialist paediatric centres (SPCs).^8–11^ In the current climate in which laparoscopic expertise continues to increase among adult general surgeons, it is logical that this expertise can be applied to paediatric patients under their care, thus allowing children to benefit from the well-recognised advantages of minimally invasive surgery for the most common paediatric surgical emergency.^4,5,9^

Despite the well-recognised advantages of laparoscopic surgery, the uptake of LA in paediatric patients internationally is behind that of adult patients.^12,13^ Currently, there are no best practice guidelines regarding the best surgical approach for management of AA in the context of a non-SPC.^12^ However, relevant data to guide practice do exist. Recent studies and meta-analyses have reported no significant difference in IAA rates between OA and LA in children, with a significant reduction in postoperative LOS, wound infection and ileus rates in LA.^7,12,14^ Long-term studies have also demonstrated greater satisfaction regarding cosmetic outcomes following LA.^12,15^ A recent meta-analysis based on six randomised controlled trials and 33 case-control studies strongly advocated for LA over OA in children, reporting consistently superior postoperative outcomes.^16^ Despite these clear advantages, overall rates of utilisation of paediatric LA lag behind adult usage and experience both in and out of SPCs.^12^

Perceived disadvantages of LA in the paediatric population have generated some resistance to utilising minimally invasive approaches in the management of AA in children. Many surgeons have avoided LA for fear of a higher incidence of postoperative IAA compared with OA. This belief has been supported by previous meta-analyses that included data from early experiences with LA in children.^17,18^ However, subsequent studies based on more recent data have shown that IAA rates are largely equivalent between both modalities.^16^ It is likely that early experiences regarding higher rates of IAA post-LA reflect the initial learning curve with this technique, with outcomes improving with developing expertise in laparoscopic surgery. The findings of our study concur that LA currently does not pose a greater risk of IAA when compared with OA. Another frequently proposed disadvantage of LA in children is that the smaller size of the patient may make the procedure more technically challenging owing to reduced working space in the intra-abdominal cavity.^8^ This was not the case in the current study, where all procedures were successfully completed laparoscopically without intraoperative or postoperative complications. It has also been proposed that LA in children is not cost-effective, creating a requirement for expensive specialised paediatric laparoscopic equipment.^8^ However, in the current study, no additional cost was generated owing to the requirement for such equipment, because all laparoscopic cases were completed using standard adult equipment. One retrospective study reported the cost of surgery in paediatric LA being €150 higher than OA, but it is important to consider this against a shorter LOS, reduced morbidity and improved long-term satisfaction.^19^ It is also true that OA has consistently been demonstrated to have a shorter operating time than LA, but our results would suggest that accepting an increase in median operating time of less than 10min can lead to significantly improved postoperative outcomes. Finally, it has been debated as to whether multiple laparoscopic port sites truly offer a postoperative recovery benefit compared with a small Lanz incision. In the current study, the LA cohort experienced reduced postoperative pain scores, less postoperative opioid use and an earlier discharge from hospital, all suggesting that LA facilitates a quicker recovery compared with OA.

### Study limitations

The current study is not without limitations. It is retrospective in nature, conducted in a single centre, involves a relatively modest cohort of patients and lacks long-term follow-up. It is also important to note that the laparoscopic cohort tended towards being slightly older and of greater weight than the open cohort, which may suggest that the former group offered more favourable conditions for minimally invasive surgery; however, these differences did not achieve statistical significance. Nonetheless, the findings are of importance. Although many studies have examined outcomes between LA and OA in paediatric patients, few have specifically looked at this when performed by adult general surgeons outside paediatric specialist practice. This is an important consideration, because it has been demonstrated that the majority of appendicectomies are performed by adult general surgeons outside SPCs, and thus these results reflect the reality of current practice. This study also specifically examined patients under the age of 13, whereas other recent studies have included patients either under the age of 16 or 18.^6–10,12,16^ This distinction was made to focus on children who were smaller in size and thus the challenges posed by laparoscopy in a smaller intra-abdominal cavity would be most apparent. Our results suggest that, even with the challenge posed by a reduced working space for laparoscopic surgery, LA is nonetheless safe and feasible.

## Conclusions

LA can be safely delivered by adult general surgeons outside SPCs to a younger paediatric population, with numerous advantages when compared with OA. A statistically significant difference in postoperative LOS, postoperative complication rate, 30-day readmission rate and negative appendicectomy rate was observed in the laparoscopic cohort. No patients in the LA group required conversion to open surgery, suggesting the feasibility of this approach. The clear benefits of LA compared with OA in this cohort demonstrated by the current study are of importance in a context in which the majority of paediatric appendicectomies are performed by adult general surgeons.

## Data availability and code availability

The data that support the findings of this study are available from the corresponding author upon reasonable request. Data analysis was performed using the software package SPSS (SPSS Inc, Chicago, IL).
